# Is elevated blood glucose at admission associated with poor outcomes in hospitalized COVID-19 patients?

**DOI:** 10.20945/2359-3997000000649

**Published:** 2023-06-19

**Authors:** Mariana Barbosa, Juliana Marques-Sá, Carla Carvalho, Vera Fernandes

**Affiliations:** 1 Hospital de Braga Serviço de Endocrinologia Braga Portugal Serviço de Endocrinologia, Hospital de Braga, Braga, Portugal; 2 Universidade do Minho Escola de Medicina Braga Portugal Escola de Medicina, Universidade do Minho, Braga, Portugal

**Keywords:** Hyperglycemia, SARS-CoV-2, complications, prognosis

## Abstract

**Objective:**

Hyperglycemia has been suggested as a risk factor for poor outcomes in coronavirus disease 2019 (COVID-19). The aim of our work was to evaluate the association between blood glucose levels at admission (BGA) and disease outcomes in hospitalized COVID-19 patients.

**Subjects and methods:**

Retrospective study including all adult COVID-19 patients admitted to a Portuguese hospital from March to August 2020 with BGA measurement. Subjects were categorized into two groups: BGA < 140 mg/dL and ≥ 140 mg/dL. Statistical analysis was performed using SPSSv26^®^ (significance defined as *p* < 0.05).

**Results:**

We included 202 patients: median age 74 (60-86) years; 43.1% female; 31.2% with diabetes. The median BGA was 130.5 (108-158) mg/dL. When compared to normoglycemic, patients with BGA ≥ 140 mg/dL were older (*p* = 0.013), more vaccinated for *influenza* (*p* = 0.025) and had more comorbidities (hypertension, heart failure and peripheral arterial disease, *p* < 0.05). The last group presented higher leucocyte and neutrophile count, higher procalcitonin and prothrombin time, and lower lymphocyte count. Concerning prognosis, BGA ≥ 140 mg/dL was associated with higher rates of mechanical ventilation requirement and intensive care unit admission (*p* < 0.001), shock (*p* = 0.011), in-hospital mortality (*p* = 0.022) and 30-day mortality (*p* = 0.037). Considering only non-diabetic patients (n = 139), those with hyperglycemia presented higher rates of severity indicators (polypnea, SatO_2_ ≤ 93% and PaO_2_/FiO_2_ ≤ 300) and an association with poor outcomes was also found, namely mechanical ventilation requirement and in-hospital/30-day mortality (*p* < 0.05).

**Conclusion:**

Hyperglycemia at admission was associated with poor outcomes in COVID-19 patients, even in those without known pre-existing diabetes. Glycemic testing should be recommended for all COVID-19 patients.

## INTRODUCTION

Coronavirus disease 2019 (COVID-19), caused by a coronavirus associated with severe acute respiratory distress syndrome 2 (SARS-CoV-2), rapidly evolved into a global crisis and was declared a pandemic on March 11, 2020, by the World Health Organization (WHO) (1).

The high rate of spread of the disease, its various clinical manifestations, as well as the associated morbidity and mortality, quickly raised interest in identifying factors influencing the risk of infection and disease prognosis. Available data showed that most cases of severe illness and death occurred in older patients with comorbidities including hypertension, diabetes mellitus (DM), coronary artery disease (CAD), chronic pulmonary disease (CPD), chronic kidney disease (CKD) and malignant neoplasms (2,3).

In fact, several studies have shown that subjects with DM were more likely to require mechanical ventilation, be admitted to intensive care units (ICU) and present higher mortality. Thus, DM can be considered a risk factor for worst prognosis and death by COVID-19 (4-8). Furthermore, in a Chinese study, diabetic patients with good glycemic control had lower mortality rates compared with those with poorer control (4).

Previous reports have demonstrated that stress hyperglycemia drives an exaggerated inflammatory response in critically ill individuals (9). In fact, recent studies have suggested that hyperglycemia at hospital admission, regardless of the diagnosis of DM, seems to conduct a worse prognosis and to increase the risk of death in patients hospitalized with COVID-19 (7,10,11). Furthermore, an Italian study found that higher glucose levels at admission were associated with the severity of COVID-19, with a stronger association among patients without as compared to those with pre-existing diabetes (12). In a pooled analysis and meta-summary of literature, Sachdeva and cols. concluded that hyperglycemia was a significant finding in patients with COVID-19 and suggested that it could be used as a prognostic marker to stratify patients based on risk for severe disease (13). However, data on this matter is scarce.

The aim of our study was to evaluate the association between blood glucose levels at admission (BGA) and disease outcomes (severity, complications, and mortality) in a population of hospitalized patients with COVID-19.

## SUBJECTS AND METHODS

### Study design and population

We present an observational and retrospective study including all adults with laboratorial confirmation of SARS-CoV-2 infection in samples from the upper or lower respiratory tract, admitted to a tertiary Portuguese hospital between March and August 2020. An initial sample of 252 patients met these criteria.

**Figure 1:**
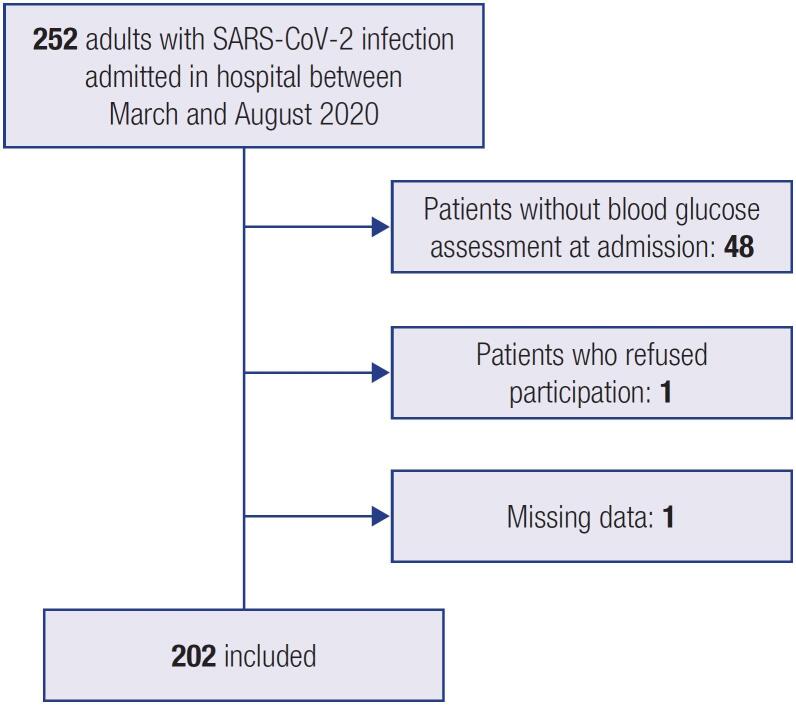
Flowchart of the study population and sample size for the final analysis.

All patients (or relatives in case of death) who met the criteria for inclusion in our study were notified by post office letters about the data collection and provided with all contact information they could use to refuse the participation in the study if intended. One patient refused participation (n = 1), while another patient with incomplete clinical records was excluded (n = 1). Additionally, subjects without blood glucose assessments at admission (n = 48) were also excluded. Thus, we obtained a final sample of 202 patients (Figure 1).

We compared patients with BGA (n = 202) and without BGA (n = 48) regarding age, gender, residence, autonomy, body mass index (BMI), smoking, alcoholism, vaccine status and medical comorbidities. We did not find any statistically significant difference, which excludes the presence of selection bias in our sample.

We collected data from electronic medical records, including demographic, anthropometric, clinical, laboratorial and radiological variables. We assessed autonomy using the Barthel index. We included BMI if assessed during hospitalization or within a maximum period of 6 months prior to admission.

We collected data regarding comorbidities found to be associated with a greater severity of COVID-19 (DM, obesity, smoking, hypertension, cardiovascular and cerebrovascular disease, CPD and malignancy) (14,15) as well those frequently reported in COVID-19 hospitalized patients [chronic liver disease (CHD) and CKD] (2,3).

The clinical manifestations of COVID-19 were considered when self-reported by patients and/or objectifiable.

We used the severity criteria established by the National Health Commission and State Administration of Traditional Chinese Medicine on March 3, 2020, to stratify the severity of disease (16). Thus, the disease was classified as severe in the presence of at least one of the following criteria: polypnea (respiratory rate ≥ 30 cpm); O_2_ saturation (SaO_2_) ≤ 93% or PaO_2_/FiO_2_ ratio ≤ 300. The severity of radiologic findings on chest computed tomography (CT) was obtained by consulting the imaging reports.

Hospital admission was considered as the 24-hour period since the first medical contact, so only laboratory values from this period were included. In addition to blood glucose at admission, we collected lab data identified as frequently altered in previous studies [hemoglobin, white blood cells (WBC), neutrophils, lymphocytes, C-reactive protein (CRP), ferritin, procalcitonin, lactate dehydrogenase (LDH), creatinine, prothrombin time (TP), albumin, aspartate aminotransferase (AST) and alanine aminotransferase (ALT), D-dimers and fibrinogen] (2,15).

In-hospital complications included in this study [acute respiratory distress syndrome (ARDS), shock, heart failure (HF), arrhythmias, stroke, acute kidney injury (AKI), bacterial superinfection, and nosocomial infection] were selected based on the available literature (2,15). ARDS was defined as acute respiratory failure (RF) accompanied by bilateral infiltrates in an imaging study in the absence of congestive HF, other forms of volume overload, pulmonary atelectasis, or nodules (17).

We divided the sample into 2 groups according to BGA. We used a cutoff point of 140 mg/dL, given the standard glycemic targets applied to hospitalized patients (18,19).

We ensured the confidentiality and anonymity of all participants and conducted the study according to a protocol properly approved by the local ethics committee (reference 87_2020).

### Statistical analysis

We performed statistical analysis using the IBM Statistical Package for the Social Sciences^®^ (SPSS), version 26.0 (IBM Corp., Armonk, NY, USA).

Categorical variables were described by the absolute and relative frequencies. For continuous variables, we assessed normality using the Kolmogorov-Smirnov and Shapiro-Wilk tests as well as evaluation of histograms. Normal continuous variables were described using mean and standard deviation while non-normal continuous variables were described using the median, 25th and 75th percentiles.

We used the χ2 test and Fisher exact test to assess the association between categorical variables. To compare continuous variables between two independent groups, we applied the t test for independent samples (*t*) or the Mann-Whitney test (U).

Models of binary logistic regression were used to better understand the impact of BGA on non-diabetic patients prognosis.

We used a 95% confidence interval and considered a result statistically significant if *p* < 0.05.

## RESULTS

An initial sample of 252 patients met the inclusion criteria of our study, as we stated above. We excluded 50 patients for the reasons previously stated in the Methods section; thus, we obtained a final sample of 202 patients (Figure 1).

We summarize the descriptive characterization of our sample in Table 1. The median age of included patients was 74 (60-86) years, and more than half of them (56.9%) were male. The mean BMI was 29.5 ± 5.7 kg/m^2^, and 78.6% of patients were overweight or obese. Regarding vaccination status, 35.5% were vaccinated against seasonal *influenza* and 14.5% had an anti-pneumococcal vaccine (1.6% with complete scheme). Most patients (84.7%) had at least one comorbidity. The most frequent comorbidity was hypertension (59.9%), followed by DM (31.2%).

**Table 1:** Characterization of the population

		n	%
**Sociodemographic data**			
Age (years) (n = 202)			
	<65	68	33.7
	65-79	54	26.7
	≥80	80	39.6
Gender (n = 202)			
	Female	87	43.1
	Male	115	56.9
Residence (n = 191)			
	Home	138	72.3
	Nursing home	53	27.7
Autonomy (n = 190)			
	Autonomous	116	61.1
	Dependent	74	38.9
BMI (kg/m^2^) (n = 42)			
	<25	9	21.4
	25-29.9	13	31.0
	≥30	20	47.6
Smoking (n = 31)		22	71.0
Alcoholism (n = 7)		6	85.7
Anti-influenza vaccine (n = 124)		44	35.5
Pneumococcal vaccine (n = 124)		18	14.5
**Medical comorbidities**			
Hypertension (n = 202)		121	59.9
HF (n = 202)		26	12.9
CAD (n = 202)		19	4.5
PAD (n = 202)		9	4.5
Cerebrovascular disease (n = 202)		22	10.9
CPD (n = 202)		34	16.8
CHD (n = 202)		3	1.5
CKD (n = 202)		21	10.4
Malignant neoplasm (n = 202)		33	16.3
DM (n = 202)		63	31.2
T1DM		2	0.1
**Symptoms**			
	Fever (n = 202)	127	62.9
	Cough (n = 202)	103	51.0
	Dyspnea (n = 160)	86	53.8
	Chest pain (n = 160)	21	13.1
	Fatigue (n = 160)	39	24.4
	Diarrhea (n = 202)	22	10.9
	Nausea (n = 160)	12	7.5
	Vomiting (n = 202)	17	8.4
	Anorexia (n = 160)	26	16.3
	Headache (n = 160)	18	11.3
	Altered mental state (n = 202)	19	9.4
	Asymptomatic (n = 202)	20	9.9

BMI: body mass index; HF: heart failure; CAD: coronary artery disease; PAD: peripheral arterial disease; CPD: chronic pulmonary disease; CHD: chronic hepatic disease; CKD: chronic kidney disease; DM: diabetes mellitus; T1DM: type 1 diabetes mellitus.

Sixty-nine percent of patients had severe disease on admission, presenting with at least one severity criterion. Mechanical ventilation was required in 23.8% of patients. In almost half of these (n = 22), invasive ventilation and ICU admission were required. The most frequent complications during hospitalization included ARDS (60.6%) and bacterial superinfection (22.3%). Nearly 29% of patients died during hospitalization, and the 30-day mortality rate was 31.2% (Table 2). The median length of stay was 11 (6.8-21.3) days.

**Table 2:** Disease severity and associated complications

		n	%
**Severity criteria**			
	Polypnea (n = 159)	75	47.2
	SatO_2_≤ 93% (n = 184)	112	60.9
	PaO_2_/FiO_2_≤ 300 (n = 184)	116	63.0
**CT Findings**			
Evidence of infection (n = 111)		99	89.2
Severity of findings (n = 60)			
	Mild	4	6.7
	Mild to moderate	1	1.7
	Moderate	29	48.3
	Moderate to severe	11	18.3
	Severe	15	25.0
**Complications**			
	RF (n = 201)	150	74.6
	Mechanical ventilation requirement (n = 202)	48	23.8
	Mechanical ventilation requirement	26	12.9
	Non-invasive ventilation	26	12.9
	Invasive ventilation	22	10.9
	ICU admission (n = 202)	22	10.9
	ARDS (n = 188)	114	60.6
	Shock (n = 199)	12	6.0
	Decompensated HF (n = 202)	9	4.5
	Arrhythmia (n = 202)	16	7.9
	Stroke (n = 202)	2	1.0
	AKI (n = 202)	28	13.9
	Bacterial superinfection (n = 202)	45	22.3
	Nosocomial infection (n = 202)	34	16.8
	In-hospital mortality (n = 202)	59	29.2
	30 days mortality (n = 202)	63	31.2

SatO_2_: saturation of oxygen; PaO_2_/FiO_2_: ratio of arterial oxygen partial pressure to fractional inspired oxygen; CT: computed tomography; RF: respiratory failure; ICU: intensive care unit; ARDS: acute respiratory distress syndrome; HF: heart failure; AKI: acute kidney injury.

### Blood glucose at admission

The median BGA was 130.5 (108-158) mg/dL. At admission, 78 patients (38.6%) had blood glucose levels ≥ 140 mg/dL. When compared to normoglycemic, patients with BGA ≥ 140 mg/dL were older [78 (65-90) *vs.* 70 (59-85) years, *p* = 0.013], more vaccinated for seasonal *influenza* (50% *vs.* 29.1%, *p* = 0.025), and had more comorbidities - hypertension (74.4% *vs.* 50.8%, *p* = 0.001), HF (20.5% *vs.* 8.1%, *p* = 0.01) and peripheral arterial disease [PAD] (9% *vs.* 1.6%, *p* = 0.029) (Table 3). Regarding COVID-19 symptoms, these patients presented with significantly more cough (57.3%, *vs.* 41%, *p* = 0.025) and altered mental state (15.4% *vs.* 5.6%, *p* = 0.021). They were also more polypneic at hospital admission (60.3% *vs.* 39.6%, *p* = 0.012).

**Table 3:** Comparative analysis between groups according to blood glucose values at admission

		Blood Glucose < 140 mg/dL (n = 124)	Blood Glucose ≥ 140 mg/dL (n = 78)	^p^
Age (years) (n = 202)				**0.018**
	<65	51 (41.1)	17 (21.8)	
	65-79	30 (24.2)	24 (30.8)	
	≥80	43 (34.7)	37 (47.4)	
Age (years) (n = 202)		70 (59-85)	78 (65-90)	**0.013**
Gender (n = 202)				0.862
	Female	54 (43.5)	33 (42.3)	
Residence (n = 191)				0.744
	Home	85 (71.4)	53 (73.6)	
	Nursing home	34 (28.6)	19 (26.4)	
Autonomy (n = 190)				0.225
	Autonomous	76 (64.4)	40 (55.6)	
	Dependent	42 (35.6)	32 (44.4)	
BMI (kg/m^2^) (n = 42)				–
	<25	4 (21.1)	5 (21.7)	
	25-29.9	8 (42.1)	5 (21.7)	
	≥30	7 (36.8)	13 (56.5)	
BMI (kg/m^2^) (n = 42)		29.4 ± 6.3	29.6 ± 5.4	0.897
Smoking (n = 31)		14 (73.7)	8 (66.7)	0.704*
Alcoholism (n = 7)		4 (100)	2 (66.7)	0.429*
Anti-influenza vaccine (n = 124)		25 (29.1)	19 (50)	**0.025**
Pneumococcal vaccine (n = 124)		12 (14)	6 (15.8)	0.789
Hypertension (n = 202)		63 (50.8)	58 (74.4)	**0.001**
HF (n = 202)		10 (8.1)	16 (20.5)	**0.01**
CAD (n = 202)		8 (6.5)	11 (14.1)	0.07
PAD (n = 202)		2 (1.6)	7 (9)	0.029*
Cerebrovascular disease (n = 202)		15 (12.1)	7 (9)	0.488
CPD (n = 202)		21 (16.9)	13 (16.7)	0.96
CHD (n = 202)		1 (0.8)	2 (2.6)	0.56*
CKD (n = 202)		11 (8.9)	10 (12.8)	0.371
Malignant neoplasm (n = 202)		25 (20.2)	8 (10.3)	0.064
DM (n = 202)		19 (15.3)	44 (56.4)	**<0.001**
**Symptoms**				
Symptom duration (days)		7 (3-9)	5 (1-8)	0.191
	Fever (n = 202)	82 (66.1)	45 (57.7)	0.227
	Cough (n = 202)	71 (57.3)	32 (41)	**0.025**
	Dyspnea (n = 160)	51 (51)	35 (58.3)	0.368
	Chest pain (n = 160)	14 (14)	7 (11.7)	0.672
	Fatigue (n = 160)	25 (25)	14 (23.3)	0.812
	Diarrhea (n = 202)	12 (9.7)	10 (12.8)	0.485
	Nausea	7 (7)	5 (8.3)	0.764*
	Vomiting (n = 202)	11 (8.9)	6 (7.7)	0.769
	Anorexia (n = 160)	20 (20)	6 (10)	0.097
	Headache (n = 160)	15 (15)	3 (5)	0.053
	Altered mental state (n = 202)	7 (5.6)	12 (15.4)	**0.021**
	Asymptomatic (n = 202)	11 (8.9)	9 (11.5)	0.537
**Severity criteria**				
	Polypnea (n = 159)	40 (39.6)	35 (60.3)	**0.012**
	SatO_2_ ≤ 93% (n = 184)	64 (56.6)	48 (67.6)	0.138
	PaO_2_/FiO_2_ ≤ 300 (n = 184)	66 (58.9)	50 (69.4)	0.149
	Severe Disease (at least one criteria)	83 (74.1)	56 (86.3)	0.198
**Laboratory data on admission**				
	Hemoglobin (g/dL) (n = 200)	13.1 (12.1-14.6)	12.8 (11.4-13.7)	**0.029**
	WBC (x10^3^/μL) (n = 199)	6.5 (4.9-9.1)	8.3 (5.3-12.7)	**0.010**
	Neutrophils (x10^3^/μL) (n = 198)	5.2 (3.2-7.6)	7.2 (3.8-10.6)	**0.012**
	Lymphocytes (x10^3^/μL) (n = 198)	1 (0.7-1.3)	0.8 (0.6-1.2)	**0.024**
	CRP (mg/dL) (n = 198)	99.2 (41.8-169.1)	102.7 (49.2-169.6)	0.855
	Ferritin (ng/mL) (n = 63)	728.5 (263.5-1213.5)	449 (244-1058)	0.574
	Procalcitonin (ng/mL) (n =51)	0.1 (0.1-0.4)	0.3 (0.1-1.8)	**0.044**
	LDH (U/L) (n = 128)	311 (230-428)	300 (231-489)	0.798
	Creatinine (mg/dL) (n = 199)	0.9 (0.7-1.2)	1.1 (0.8-1.4)	**0.006**
	PT (s) (n = 120)	11.9 (11.3-12.8)	12.5 (11.5-16.3)	**0.019**
	Albumin (g/dL) (n = 24)	3.5 ± 0.5	3.5 ± 0.6	0.910
	AST (U/L) (n = 152)	38 (26-64)	40 (26-54)	0.89
	ALT (U/L) (n = 159)	31 (23-47)	30 (21-45)	0.316
	D-dimer (ng/mL) (n = 86)	990 (651-2588)	1063 (788-2233)	0.673
	Fibrinogen (mg/dL) (n = 24)	566.4 ± 154.6	541.4 ± 210.1	0.739
**CT Findings**				
	Evidence of infection (n = 111)	62 (92.5)	37 (84.1)	0.214*
Severity of findings (n = 60) Mild		3 (7.7)	1 (4.8)	–
	Mild to moderate	0	1 (4.8)	
	Moderate	20 (51.3)	9 (42.9)	
	Moderate to severe	9 (23.1)	2 (9.5)	
	Severe	7 (17.9)	8 (38.1)	
**Complications**				
RF(n = 201)		85 (69.1)	65 (83.3)	**0.024**
**Ventilation requirement**				
	No	106 (85.5)	48 (61.5)	**<0.001**
	Non-invasive ventilation	12 (9.7)	14 (17.9)	
	Invasive ventilation	6 (4.8)	16 (20.5)	
ICU admission (n = 202)		6 (4.8)	16 (20.5)	**<0.001**
ARDS (n = 188)		64 (56.1)	50 (67.6)	0.117
Shock (n = 199)		3 (2.4)	9 (12)	0.011*
Decompensated HF (n = 202)		1 (0.8)	8 (10.3)	0.002*
Arrhythmia (n = 202)		6 (4.8)	10 (12.8)	**0.041**
Stroke (n = 202)		1 (0.8)	1 (1.3)	1.000*
AKI (n = 202)		11 (8.9)	17 (21.8)	**0.01**
Bacterial superinfection (n = 202)		21 (16.9)	24 (30.8)	**0.021**
Nosocomial infection (n = 202)		20 (16.1)	14 (17.9)	0.736
In-hospital mortality(n = 202)		29 (23.4)	30 (38.5)	**0.022**
30 days mortality (n = 202)		32 (25.8)	31 (39.7)	**0.037**
Length of hospital stay (n = 202)		10 (6.3-18)	14 (6.8-29.3)	0.062

* Fisher’s exact test.

BMI: body mass index; HF: heart failure; CAD: coronary artery disease; PAD: peripheral arterial disease; CPD: chronic pulmonary disease; CHD: chronic hepatic disease; CKD: chronic kidney disease; DM: diabetes mellitus; SatO_2_: saturation of oxygen; PaO_2_/FiO_2_: ratio of arterial oxygen partial pressure to fractional inspired oxygen; WBC: white blood cells; CRP: C-reactive protein; LDH: lactic acid dehydrogenase; PT: prothrombin time; AST: aspartate transaminase; ALT: alanine aminotransferase; CT: computed tomography; RF: respiratory failure; ICU: intensive care unit; ARDS: acute respiratory distress syndrome; AKI: acute kidney injury.

Laboratory data on admission revealed that hyperglycemic patients presented significantly higher WBC levels (8.3 *vs.* 6.5 × 10^3^/μL, *p* = 0.010), neutrophils (7.2 *vs.* 5.2 × 10^3^/μL, *p* = 0.012), procalcitonin (0.3 *vs.* 0.1 ng/mL, *p* = 0.044), creatinine (1.1 *vs.* 0.9 ng/dL, *p* = 0.006), PT (12.5 *vs.* 11.9s, *p* = 0.019), and significantly lower values of hemoglobin (12.8 *vs.* 13.1 g/dL, *p* = 0.029) and lymphocytes (0.8 *vs.* 1x10^3^/μL, *p* = 0.024).

Concerning prognosis, patients with blood glucose at admission ≥ 140 mg/dL developed more RF (83.3% *vs.* 69.1%, *p* = 0.024), higher rates of mechanical ventilation (38.5% *vs.* 14.5%, *p* < 0.001) and ICU admission (20.5% *vs.* 4.8%, *p* < 0.001). Additionally, they more frequently developed other complications, namely septic shock (12% *vs.* 2.4%, *p* = 0.011), HF (10.3% *vs.* 0.8%, *p* = 0.002), AKI (21.8% *vs.* 8.9%, *p* = 0.01), and bacterial superinfection (30.8% *vs.* 16.9%, *p* = 0.021). They also presented significantly higher in-hospital mortality (38.5% *vs.* 23.4%, *p* = 0.022) and higher 30-day mortality (39.7% *vs.* 25.8%, *p* = 0.037).

The association between BGA and the variables of interest was also analyzed in the group of patients without DM to determine whether the previously described associations maintained statistical significance in the absence of DM (Table 4). Considering only non-diabetic patients (n = 139), 24.5% had BGA ≥ 140 mg/dL. There were no differences in comorbidities between BGA groups, except for age and hypertension. Nevertheless, non-diabetic patients with blood glucose ≥ 140 mg/dL (n = 34) presented higher values of WBC (8.8 *vs.* 6.7 × 10^3^/μL, *p* = 0.02), neutrophils (7.6 *vs.* 5.5 × 10^3^/μL, *p* = 0.018) and PT (14 *vs.* 12s, *p* = 0.005), and lower values of hemoglobin (12.8 *vs.* 13.3 g/dL, *p* = 0.025) and lymphocytes (0.7 *vs.* 0.9 × 10^3^/μL, *p* = 0.017). Hyperglycemic non-diabetic patients also presented higher rates of severity indicators: they were more polypneic at admission (64.3% *vs.* 38.8%, *p* = 0.019) and presented more frequently with SatO_2_ ≤ 93% and PaO_2_/FiO_2_ ≤ 300. Additionally, they developed more RF (88.2% *vs.* 65.4%, *p* = 0.011), a greater need for mechanical ventilation (38.2% *vs.* 13.3%, *p* = 0.001) and higher in-hospital mortality (47.1 % *vs.* 24.8%, *p* = 0.014), as well as higher 30-day mortality (47.1% *vs.* 25.7%, *p* = 0.019).

**Table 4:** Comparative analysis between groups according to blood glucose values at admission in patients without diabetes

		Blood Glucose < 140 mg/dL (n = 105)	Blood Glucose ≥ 140 mg/dL (n = 34)	^p^
Age (years) (n = 139)				0.030
	<65	46 (43.8)	7 (20.6)	
	65-79	24 (22.9)	8 (23.5)	
	≥80	35 (33.3)	19 (55.9)	
Age (years) (n = 139)		67 (56-84)	84 (65-91)	0.006
Gender (n = 139)				0.821
	Female	44 (41.9)	15 (44.1)	
	Male	61 (58.1)	19 (55.9)	
Residence (n = 133)				0.800
	Home	72 (72)	23 (69.7)	
	Nursing home	28 (28)	10 (30.3)	
Autonomy (n = 130)				0.382
	Autonomous	66 (66.7)	18 (58.1)	
	Dependent	33 (33.3)	13 (41.9)	
BMI (kg/m^2^) (n = 17)				–
	<25	4 (33.3)	2 (40)	
	25-29.9	3 (25)	1 (20)	
	≥30	5 (41.7)	2 (40)	
BMI (kg/m^2^) (n = 17)		28.3 ± 5.56	26.1 ± 5.46	0.463
Smoking (n = 24)		13 (72.2)	5 (83.3)	1.000*
Alcoholism (n = 6)		4 (100)	2 (100)	–
Anti-influenza vaccine (n = 88)		15 (20.5)	5 (33.3)	0.316*
Pneumococcal vaccine (n = 88)		8 (11)	2 (13.3)	0.677*
Hypertension (n = 139)		47 (44.8)	22 (64.7)	0.043
HF (n = 139)		6 (5.7)	6 (17.6)	0.071*
CAD (n = 139)		6 (5.7)	5 (14.7)	0.137*
PAD (n = 139)		2 (1.9)	0	1.000*
Cerebrovascular disease (n = 139)		10 (9.5)	4 (11.8)	0.746*
CPD (n = 139)		16 (15.2)	6 (17.6)	0.738
CHD (n = 139)		1 (1)	0	1.000*
CRD (n = 139)		7 (6.7)	1 (2.9)	0.679*
Malignant neoplasm (n = 139)		23 (21.9)	4 (11.8)	0.194
**Symptoms**				
Symptom duration(days) (n = 108)		7 (3-9)	7 (4.5-1)	0.376
	Fever (n = 139)	71 (67.6)	18 (59.2)	0.179
	Cough (n = 139)	59 (56.2)	12 (35.3)	0.055
	Dyspnea (n = 112)	45 (52.3)	16 (61.5)	0.547
	Chest pain (n = 112)	13 (15.1)	3 (11.5)	0.760*
	Fatigue (n = 112)	19 (22.1)	4 (15.4)	0.642
	Diarrhea (n = 139)	11 (10.5)	5 (14.7)	0.540*
	Nausea (n = 112)	6 (7)	2 (7.7)	1.000*
	Vomiting (n = 139)	10 (9.5)	4 (11.8)	0.746
	Anorexia (n = 112)	16 (18.6)	2 (7.7)	0.235*
	Headache (n = 112)	14 (16.3)	2 (7.7)	0.353*
	Altered mental state (n = 139)	7 (6.7)	6 (17.6)	0.085*
	Asymptomatic (n = 139)	11 (10.5)	6 (17.6)	0.365*
**Severity criteria**				
Polypnea (n = 113)		33 (38.8)	18 (64.3)	**0.019**
SatO_2_ ≤ 93% (n = 127)		52 (54.2)	23 (74.2)	**0.049**
PaO_2_/FiO_2_ ≤ 300 (n = 125)		52 (55.9)	25 (78.1)	**0.026**
Severe disease (at least one criteria) (n = 124)		68 (73.1)	26 (89.3)	0.333
**Laboratory data on admission**				
Hemoglobin (g/dL) (n = 137)		13.3 (12.3-14.6)	12.8 (11.5-13.5)	**0.025**
BWC(x10^3^/μL) (n = 137)		6.7 (4.9-9.3)	8.8 (6.5-12.7)	**0.02**
Neutrophils (x10^3^/μL) (n = 135)		5.5 (3.3-7.9)	7.6 (5.1-10.6)	**0.018**
Lymphocytes (x10^3^/μL) (n = 135)		0.9 (0.7-1.3)	0.7 (0.6-1.03)	**0.017**
RCP (mg/dL) (n = 136)		100.7 (41.6-179.7)	128.2 (48.2-167.9)	0.931
Ferritin (ng/mL) (n = 39)		741.5 (297.3-1313.8)	716 (308-2931)	0.512
Procalcitonin (ng/mL) (n = 35)		0.2 (0.1-0.5)	0.3 (0.1-1.9)	0.226
LDH (U/L) (n = 87)		315 (253-428)	331 (222.5-549)	0.468
Creatinine (mg/dL) (n = 137)		0.9 (0.6-1)	1 (0.8-1.3)	0.078
PT (s) (n = 79)		12 (11-13)	14 (12-20)	**0.005**
Albumin (g/dL) (n = 15)		3.5 ± 0.5	3 ± 0.6	0.221
AST (U/L) (n = 100)		39 (27-64)	44 (25-60.5)	0.842
ALT (U/L) (n = 104)		32 (23-51)	31 (24-38)	0.360
D-dimer (ng/mL) (n = 58)		1252.5 (749.8-3593.5)	1568 (955-2799.3)	0.669
Fibrinogen (mg/dL) (n = 15)		560.6 ± 168.9	475.8 ± 226	0.443
**CT Findings**				
Evidence of infection (n = 74)		54 (91.5)	13 (86.7)	0.624*
Severity of findings (n = 42)				–
	Mild	1 (2.9)	0	
	Moderate	18 (52.9)	2 (25)	
	Moderate to severe	8 (23.5)	1 (12.5)	
	Severe	7 (20.6)	5 (62.5)	
**Complications**				
	RF (n = 138)	68 (65.4)	30 (88.2)	**0.011**
	Ventilation Requirement (n = 139)	14 (13.3)	13 (38.2)	**0.001**
	ICU admission (n = 139)	4 (3.8)	4 (11.8)	0.100*
	ARDS (n = 129)	53 (54.6)	21 (65.6)	0.276
	Shock (n = 139)	2 (1.9)	3 (8.8)	0.094*
	Decompensated HF (n = 139)	1 (1)	2 (5.9)	0.148*
	Arrythmia (n = 139)	5 (4.8)	5 (14.7)	0.065*
	Stroke (n = 139)	1 (1)	1 (2.9)	0.431*
	AKI (n = 139)	9 (8.6)	7 (20.6)	0.068*
	Bacterial superinfection (n = 139)	18 (17.1)	11 (32.4)	0.058
	Nosocomial infection (n = 139)	15 (14.3)	9 (26.5)	0.102
	In hospital mortality (n = 139)	26 (24.8)	16 (47.1)	**0.014**
	30 days mortality (n = 139)	27 (25.7)	16 (47.1)	**0.019**
	Length of hospital stay (days) (n = 139)	10 (6-18)	11 (6-24.5)	0.545

* Fisher’s exact test.

BMI: body mass index; HF: heart failure; CAD: coronary artery disease; PAD: peripheral arterial disease; CPD: chronic pulmonary disease; CHD: chronic hepatic disease; CKD: chronic kidney disease; SatO_2_: saturation of oxygen; PaO_2_/FiO_2_: ratio of arterial oxygen partial pressure to fractional inspired oxygen; WBC: white blood cells; CRP: C-reactive protein; LDH: lactic acid dehydrogenase; PT: prothrombin time; AST: aspartate transaminase; ALT: alanine aminotransferase; CT: computed tomography; RF: respiratory failure; ICU: intensive care unit; ARDS: acute respiratory distress syndrome; AKI: acute kidney injury.

To better assess the role of hyperglycemia in non-diabetic patients, we used binary logistic regression models for each complication adjusted to age, presence of hypertension, and BGA group at admission (Table 5). The inclusion of these variables was based on the statistically significant differences found in patients without diabetes compared according to BGA (Table 4). We found that BGA > 140 mg/dL at admission was an independent predictor factor of RF and the need for mechanical ventilation. On the other hand, BGA > 140 mg/dL at admission did not represent an independent predictor factor of in-hospital mortality or 30-day mortality. We also found that age was an independent predictor factor of RF, in-hospital, and 30-day mortality. Hypertension was an independent predictor factor of the need for mechanical ventilation.

**Table 5:** Results of binary logistic regressions models for complications

Complication		OR	95% CI
Respiratory failure			
	BGA > 140 mg/dL	3.241	1.009-10.414
	Age	1.046	1.018-1.074
	Hypertension	0.905	0.380-2.155
Need of mechanical ventilation			
	BGA > 140 mg/dL	3.167	1.214-8.265
	Age	1.009	0.979-1.041
	Hypertension	4.779	1.559-14.653
In-hospital mortality			
	BGA > 140 mg/dL	1.919	0.751-4.903
	Age	1.080	1.045-1.116
	Hypertension	0.672	0.268-1.683
30-day mortality			
	BGA > 140 mg/dL	1.831	0.711-4.714
	Age	1.084	1.049-1.121
	Hypertension	0.584	0.230-1.480

BGA: blood glucose levels at admission; CI: confidence interval; OR: odds ratio.

## DISCUSSION

DM is a known risk factor for worst prognosis and death in COVID-19 patients (4-8). However, data on the specific prognostic value of hyperglycemia in COVID-19, regardless of the presence of diabetes, is less exploited in the literature. Several studies have shown that stress-induced hyperglycemia at hospital admission for acute medical or surgical illness in subjects with no history of diabetes is a worse predictor than diabetes for poor clinical outcomes and mortality (9,20-22).

Concerning our results, when compared to normoglycemic, patients with BGA ≥ 140 mg/dL were older, more vaccinated for *influenza*, and had more comorbidities, namely hypertension, HF and PAD. These whole-sample associations may be influenced by the confounding effect of diabetes diagnosis among included subjects, as diabetic patients are more likely to present these features. In fact, when we considered only non-diabetic subjects, we found no differences in comorbidities between BGA groups except for age and hypertension, which goes in line with our hypothesis.

In our sample, hyperglycemic patients presented with significantly more cough, altered mental state and polypnea at hospital admission. Hyperglycemia was also associated with more pronounced elevation of some systemic inflammatory markers and laboratorial findings associated with severe COVID-19 disease. These results are consistent with published data (23-25). On the other hand, we found no differences regarding other coagulation and inflammatory markers commonly described as altered in the literature, such as D-dimer, fibrinogen, CRP and LDH (23-25).

Regarding prognosis, in our population BGA ≥ 140 mg/dL was associated with higher rates of RF, mechanical ventilation requirement, ICU admission, in-hospital mortality and 30-day mortality. These findings are in line with previous reports. In a multicenter Spanish study involving 11,312 patients, admission hyperglycemia was associated with the requirement for mechanical ventilation and ICU admission; hyperglycemia was also an independent risk factor for mortality, after adjustment for age, diabetes, hypertension and other confounding factors (25). In a cohort study by Wu and cols., elevation of BGA was an independent risk factor for progression to critical cases/death among non-critical cases in hospitalized patients with COVID-19 (HR: 1.30, 95% CI: 1.03 to 1.63, *p* = 0.026) (10). Furthermore, a recent meta-analysis demonstrated that the hyperglycemia at admission group of subjects was more likely to have increased mortality (OR: 3.45, 95% CI: 2.26-5.26) and severe/critical complications (OR: 2.08, 95% CI: 1.45-2.99) of COVID-19 when compared with the control group (26). Moreover, hyperglycemic patients in our sample presented higher rates of septic, cardiovascular and renal complications during hospitalization. Hence, these findings suggest that hyperglycemia is probably associated with a more pronounced systemic involvement of the disease, resulting in multiorgan decompensations.

When we analyzed only patients without diabetes, we also found statistically significant differences between BGA groups in the two other disease severity criteria apart from polypnea: hyperglycemic non-diabetic subjects presented more frequently with SatO_2_ ≤ 93% and PaO_2_/FiO_2_ ≤ 300. This is in accordance with findings from Fadini and cols. stating that the association between admission hyperglycemia and adverse outcomes was mostly mediated by a worse respiratory function, with rapid pulmonary deterioration (12). Finally, in our sample, the association of hyperglycemia with poorer clinical outcomes persisted in the subgroup of patients without diabetes for RF, mechanical ventilation requirement and in-hospital/30-day mortality. Moreover, hyperglycemia at admission represented an independent predictor factor for RF and mechanical ventilation requirement. Sachdeva and cols. showed that hyperglycemia in non-diabetics was associated with a higher risk of severe/critical illness and mortality compared with those with normal BGA (13). In the multicenter retrospective study by Yang and cols., which included only COVID-19 patients without previous diagnosis of diabetes, admission fasting blood glucose was an independent predictor for 28-day mortality (11), a result that we did not find in our sample since only age was an independent predicting factor for mortality. Two recent studies also demonstrated a significant association between hyperglycemia and increased severity, mortality, and morbidity in COVID-19 hospitalized patients with and without DM, and the association was stronger among patients without DM (12,27).

The mechanism by which acute hyperglycemia drives the progression of COVID-19 remains under investigation, and several hypotheses have been suggested (28). Previous research has shown that hyperglycemia can cause impairment in innate immunity, leading to an increased risk of infection (29). Recently, Fadini and cols. suggested that, in COVID-19 patients, admission glucose association with adverse outcomes was mostly mediated by worse respiratory function, as mentioned before (12). Hyperglycemia also generates reactive oxygen species and induces oxidative stress, leading to endothelial dysfunction and potentially enabling further pulmonary microangiopathy (9,28). Moreover, glucose has pro-inflammatory effects, and this milieu of heightened inflammation can possibly contribute to the cytokine storm witnessed in COVID-19 patients. Additionally, sustained hyperglycemia can lead to glycosylation of angiotensin-converting enzyme 2 (ACE2) receptors; SARS-CoV-2 can bind to these receptors, and their glycosylation favors virus cellular intrusion, subsequently leading to a more widespread and severe disease (13,30).

However, a bidirectional relationship between COVID-19 and hyperglycemia has been postulated (23,28). Infection might trigger the release of proinflammatory cytokines leading to insulin resistance as well as the release of stress hormones that trigger liver glycogenolysis (9,23). Moreover, ACE2 receptors are found to be expressed in pancreatic beta-cells, providing a possible direct route for the virus to enter and damage the pancreatic islets, leading to possible insulin deficiency and subsequent hyperglycemia (23,28,31).

Our work includes a considerable number of patients representative of the first phase of the SARS-Cov-2 pandemic in the north of Portugal. To the best of our knowledge, this was the first cohort of COVID-19 hospitalized patients to provide relevant information on the specific potential role of glycemic evaluation and its association with prognostic parameters in the Portuguese population. Additionally, this study demonstrates that, in non-diabetic patients, hyperglycemia at admission is an independent predicting factor for RF and the need for mechanical ventilation. Thus, the management of these patients should change, and surveillance should be increased.

On the other hand, this study also presents some limitations: first, its retrospective character, limiting data collection strictly to the available information on medical records – namely data on BMI, a parameter with documented association with prognosis in these patients (32-34) that was available in only 42 subjects, as well as other variables with missing data that could add potential bias to the final results obtained; second, the inclusion of only hospitalized patients from a single center; and, third, the inherent limitations of the comparative analysis of our findings with published data, given the heterogeneous definitions of hyperglycemia in the literature.

In summary, BGA measurement should be recommended for all COVID-19 patients, even in those without known pre-existing diabetes. In fact, glycemic testing may be used as a simple tool to help stratify risk of COVID-19 patients for hierarchical management in clinical practice (10). Moreover, although large intervention studies are lacking, some reports have already suggested that an early and intensive intervention to optimally lower blood glucose levels might help in improving clinical outcomes (35,36). Overall, this data emphasizes the major relevance of systematic BGA evaluation in COVID-19 patients.
